# Determination of Electrode Kinetics Parameters from Dynamic Electrochemical Impedance Spectroscopy Measurements via Potential-Program Invariant Functions

**DOI:** 10.1021/acs.jpclett.3c02810

**Published:** 2023-11-17

**Authors:** Tamás Pajkossy

**Affiliations:** Institute of Materials and Environmental Chemistry, Research Centre for Natural Sciences, Magyar tudósok körútja 2, Budapest H-1117, Hungary

## Abstract

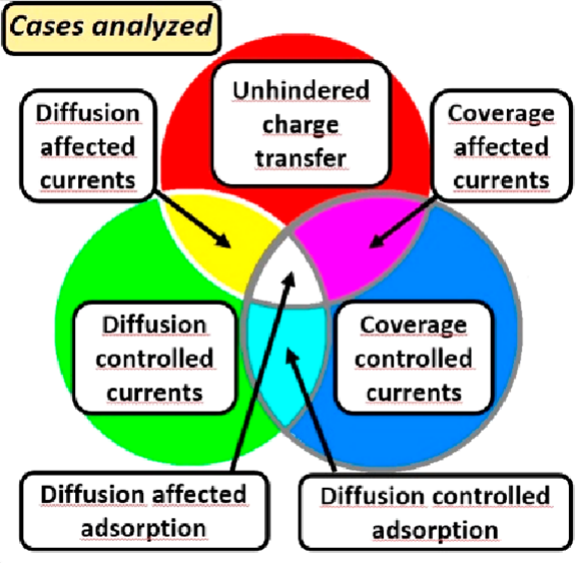

Dynamic electrochemical impedance spectroscopy, dEIS, comprises repetitive impedance spectrum measurements while slow scan-rate voltammetry is running. Its main virtue is the short measurement time, reducing the danger of contamination of the electrode surface. To further the use of dEIS, we have recently elaborated a set of theories aimed at the related data processing for three groups of fundamental electrode reactions: diffusion-affected charge transfer, charge transfer of surface-bound species, and adsorption–desorption. These theories yielded equations by which the voltammograms can be transformed to potential-program invariant forms, allowing an easy calculation of the rate coefficients; similar equations have been derived for the potential dependence of equivalent circuit parameters obtained from the impedance spectra. In this Perspective, the above derivations are condensed into a single, unified one. The theory is recommended to evaluate electrode kinetic measurements, particularly when the potential dependence of rate coefficients is under study.

Kinetics of electrode processes have always played an important role in physical chemistry just as in practical issues such as electrocatalysis, batteries, and corrosion prevention. Determination of process rates and rate coefficients, just like in other branches of chemical kinetics, has been the focus of many researchers and engineers. In the present Perspective, we introduce new evaluation techniques for the experimental technique dynamic electrochemical impedance spectroscopy, dEIS, to further its role in electrochemical kinetics in three respects.

First, dEIS is a much faster method than most sorts of voltammetry, e.g., the usual cyclic voltammetry, CV, and standard electrochemical impedance spectroscopy, EIS. During a shorter measurement time, the electrode surface has less chance to be contaminated; hence, conditions of the determination of the rates of processes are much better defined.

Second, the traditional evaluation methods are usually based on models, in which the charge transfer rate coefficients are assumed to exhibit exponential dependence on electrode potential. This constraint does not necessarily apply and therefore may bias the evaluation. Instead, in the suggested method, the potential dependence of the rate coefficients is an output—rather than an input—feature.

Third, the suggested evaluation method eliminates the irrelevant, incidental measurement parameters like scan rates and transforms the measured curves like voltammograms to standardized, potential-program invariant (PPI) forms, thereby assisting in comparing different measurements.

In general, the most fundamental experimental technique of studying electrode kinetics is to measure current–voltage curves or, to be precise, current density, *j*, against electrode potential, *E*. This can be done if the system is in a steady state; otherwise, recording dependences on time or frequency (*t* or ω, respectively) should be involved in the study. Well-established techniques exist for this task; voltammetry and impedance spectroscopy are the most widely used family of methods.^1−3^ The former method is the measurement of *j*(*t*) with changing potentials in a broad range of potentials, *E*(*t*), the function of which is named the potential program. With the aim of not restricting generality, we consider experiments not only with traditional CVs (with linear sweeps, i.e., with *E*(*t*) evenly changing in time) but also with arbitrary *E*(*t*) program voltages. For these, the term arbitrary waveform voltammetry, AWV, will be used. In contrast to AWV experiments performed in broad potential ranges, EIS is based on small-amplitude perturbations around a preset potential; it provides the correlation of the response and the perturbation in the frequency domain. Disregarding the perturbation, EIS requires the equilibrium—or at least steady state—of the electrode. Not only the potential range studied but also the usual roles of AWV and EIS are different: AWV serves mainly for the qualitative characterization of the electrochemical behavior of the system studied (e.g., to detect processes), whereas EIS is used to extract precise values of the model parameters provided we already know which model should be applied.

Electrochemical systems often are in a nonsteady state. There exist cases of transient phenomena when the interface structure changes in time, altering the reactions proceeding therein. Contaminations adsorbing onto the electrode may spoil the surface, thereby leaving a short (seconds to minutes) time window for performing impedance measurements. These are the situations when EIS should be measured as fast as possible; this can be achieved by simultaneously performing the measurement in a broad range of frequencies using typically odd-harmonic, random phase multisine perturbation^4−8^—at the expense of somewhat decreased accuracy.^9^ One special form of the multisine EIS is a combination with AWV: in what follows, we shall call it dynamic EIS, dEIS. Provided that the scan rate of the AWV and the lowermost frequency of EIS are sufficiently low and high, respectively, we get a series of “well-behaving”, i.e., Kramers–Kronig-test compatible, spectra^3^ with certain potential spacing from each other. In contrast to the commercially available, widely used methods of steady-state EIS, sEIS, and multisine EIS, dEIS is a laboratory curiosity that has been built up and used by a few electrochemist groups in the past decades; representative publications include refs (10−15).

The need and use of dEIS prompted the present author recently to reformulate the laws and equations of certain basic electrode kinetic situations for both AWV and EIS. These are the quasi-reversible reactions (using the polarography terminology), that is, when the interfacial reaction rates are finite rather than infinite. The main papers on this subject are refs (16−19). Reformulation was achieved with no *a priori* assumption on the potential dependence of the relevant rate coefficients. As a consequence, the resulting equations do not contain the *E*(*t*) potential program; AWVs recorded with any *E*(*t*) potential program (under potentiostatic or galvanostatic conditions or any type of control) can be transformed to the same potential-program invariant form. Equivalent circuit parameters calculated from dEIS measurements usually do depend on the scan rate; however, this dependence can be eliminated by a similar transformation yielding PPI values. An illustration of the main point is shown in Figure 1: the voltammogram of the diffusion-affected charge transfer with four cycles of *E*(*t*) with increasing scan rates is of complicated shapes with hysteresis (Figure 1a). However, with the transformation suggested in ref (16), we can get hysteresis-free curves, as shown in Figure 1b. This way the consequences of the (irrelevant) measurement conditions have been eliminated; all that remains are the (relevant) physicochemical functions.

**Figure 1:**
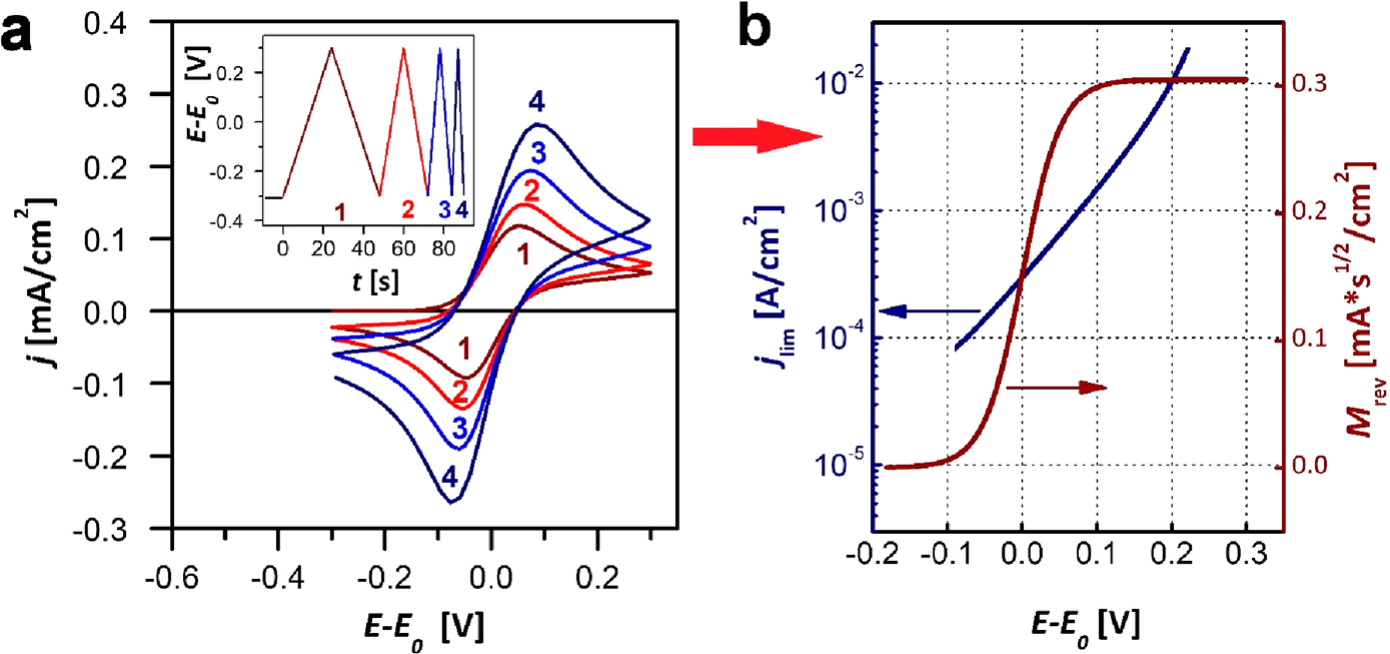
Example of the transformation of a simulated four-cycle CV. (a) With changing scan rates of diffusion-affected charge transfer electrode reaction to two potential-program invariant functions. For the potential program, see the inset. (b) *j*_lim_ and *M*_rev_ are functions characterizing charge transfer and diffusion separately; for their meaning, see the Theory discussion.

The structure of this Perspective is simple and traditional. First, the relevant equations for various cases of kinetics are derived, with a view to equations connecting AWV and EIS. We apply a formalism that yields a much more compact set of equations than that in previous papers. Later on, various properties and implications of the theory are analyzed, including an experiment by which the electron transfer rate coefficients have recently been determined. Finally, as conclusions, we advocate using the theory together with dEIS for studying electrode kinetics.

*Theory—Voltammograms*. Consider a *j*(*t*) vs *E*(*t*) voltammetry measurement of a system:
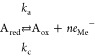
1

These species take part in an *n*-electron interfacial reaction with no detectable intermediates; both the anodic and cathodic rate coefficients, *k*_a_(*E*) and *k*_c_(*E*), respectively, are potential-dependent, in a nonspecified way (i.e., not necessarily in the usual exponential fashion). Two cases will be considered.

In the first case, both species are components of the electrolyte: the reaction is a charge transfer whose rate is hindered (affected or fully controlled) by species transport to and from the electrode surface. Effects of convection and migration are disregarded, and only diffusion is considered. Hence, this case will be shortly named with the abbreviation “diffredox”; the rate equation, expressed as current density, is assumed to be first-order for both species A_red_ and A_ox_, that is,
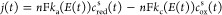
2where the concentrations are the ones in the vicinity of the surface and *F* is Faraday’s constant.

In the second case, both components are bound to the surface, and the rate equation contains the θ and (1 – θ) coverages of the two species:
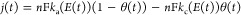
3

This case will be shortly named “surfredox”. Based on the role of the coverage in the kinetics equation, the term “coverage hindrance” will also be used.

There is a third, intermediate situation when one of the species is in the electrolyte bulk and the other is bound to the surface, like when adsorption–desorption prevails (with or without diffusional hindrance). This case, named the “adsorption case”, is analyzed later. Together, the diffredox and surfredox reaction schemes cover a fairly large family of interfacial reactions.

Time dependence of the current density, *j*(*t*), enters the picture due to the time dependences of the surface concentrations (for the diffredox case) and of coverages (for the surfredox case). Note that both eqs 2 and 3, for one particular chosen potential, ε, are of the following form:
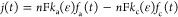
4

That is, the dependences of potential and time are separate factors. We proceed by recognizing that for both cases there exists a connection between the time-dependent *f*(*t*) values and *j*(*t*).

For the diffredox case, the Matsuda–Ayabe equation connects the surface concentrations with the flux of the diffusing particles onto the surface;^20^ see also the Supplement of ref (21). This equation is a solution of Fick’s equations for the case when both species are in an initially homogeneous electrolyte of infinite volume and the electrode surface is planar. It contains a convolution integral often called the semi-integral, *M*(*t*), of the current:^22^
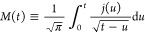
5The Matsuda–Ayabe equations for the two species are
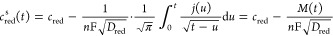
6and
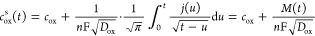
7where *c*_red_ and *c*_ox_ stand for the bulk concentrations of A_red_ and A_ox_, respectively, and the *D* values are diffusion coefficients. Substituting *c*_red_^s^(*t*) and *c*_ox_^s^(*t*) from eqs 6 and 7 into eq 2 at potential ε, we get
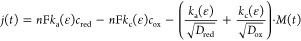
8Note the linear connection of *j*(*t*) and *M*(*t*) at constant ε potential.

For the surfredox case, the *f*(*t*) values are related to the *q*(*t*) surface charge through the coverages. Provided that the initial condition is θ(*t* ≤ 0) = 0, and hence *q*(*t* ≤ 0) = 0, then
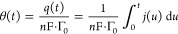
9where Γ_0_ is the sum of the surface concentrations of the two species (in mol/cm^2^ units). We note that a derivation with a more general initial condition 0 ≤ θ(*t* ≤ 0) ≤ 1 can be found in ref (19)—it has essentially the same outcome.

Substitution of θ(*t*) of eq 9 into eq 3 yields the following equation, at ε potential:
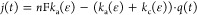
10

Note that eqs 8 and 10 are of the same linear form of
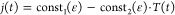
11where *T*(*t*) (“transformed current”) is the collective term for the operations of semi-integration, *M*(*t*), and of integration, *q*(*t*). In what follows, const_1_ will be denoted as *j*_lim_, with its subscript expressing that the current is of limit value when the hindrance due to diffusion or to the coverage of the surface is still negligible. Const_2_, denoted as *H*, is a combination of parameters affecting the reaction rate. This way eq 11 can be rewritten in the form
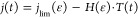
12or, after rearranging,
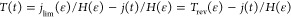
13

The subscript “rev” has a polarography terminology origin, namely, the physical meaning of *T*_rev_ is the value of *T*(*t*) when *j*(*t*) = 0; hence, it means the transformed (semi-integrated or integrated) *j*(*t*) when the charge transfer is very fast and kinetically reversible. This definition of *T*_rev_(ε) implies that
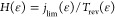
14The combination of eqs 12 and 14 yields the most compact form:
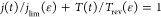
15

The definitions and constants are summarized in Table 1. Note the double meaning of *H*(ε): first, a parameter combination of the model of kinetics (third column of Table 1), and second, the ratio of intercepts (fourth column therein). For further details, see the references in the rightmost column.

**Table 1:** Constants of Equations 11 and 12 for the Two Cases

Case, *T*(*t*)	const_1_ = *j*_lim_	const_2_ = *H*	*H*	Ref
diffredox: *T*(*t*) = *M*(*t*)	*n*F*k*_a_*c*_red_ – *n*F*k*_c_*c*_ox_	(*k*_a_/√*D*_red_) + (*k*_c_/√*D*_ox_)	*j*_lim_/*M*_rev_	(16)
surfredox: *T*(*t*) = *q*(*t*)	*n*F*k*_a_	*k*_a_ + *k*_c_	*j*_lim_/*q*_rev_	(19)

The practical use of eq 12 is straightforward: we carry out a voltammetry experiment with any type of potentiostatic or galvanostatic control, with any potential program for which no current flows before the experiment [*j*(*t* ≤ 0) = 0]. During the experiment (*t* > 0), the potential should attain or cross the *E* = ε value at least two times. Various *E*(*t*) potential programs are possible, as illustrated in Figure 2, including repeated experiments with altered *E*(*t*) potential programs. All the points, taken at ε potential, plotted in the *j*(*t*) vs *T*(*t*) representation will lie on a straight line, according to eq 12 as illustrated in Figure 3. This is how the parameters of kinetics (*j*_lim_, *H*) and/or of transport or thermodynamic relevance (*M*_rev_ and *q*_rev_, respectively) can be calculated; this is how the points of the two curves of Figure 1b have been calculated from the *j*(*t*) vs *E*(*t*) data of Figure 1a. For further examples of how complete CVs (or AWVs) have been transformed, see refs (16, 18, and 19).

**Figure 2:**
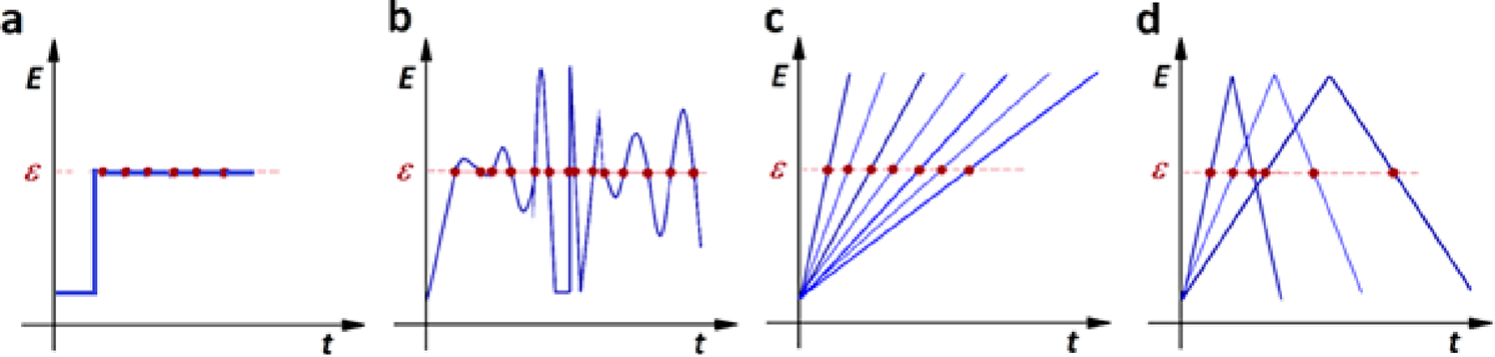
Possible potential programs of a single (a, b) or repeated (c, d) voltammetry experiment, with (a) a single potential step or (b) an arbitrary potential program or (c) repeated linear scans or (d) repeated cycles. All start from a potential where for *t* ≤ 0 either the electrolyte is homogeneous (in the diffredox case) or θ = 0 (in the surfredox case).

**Figure 3:**
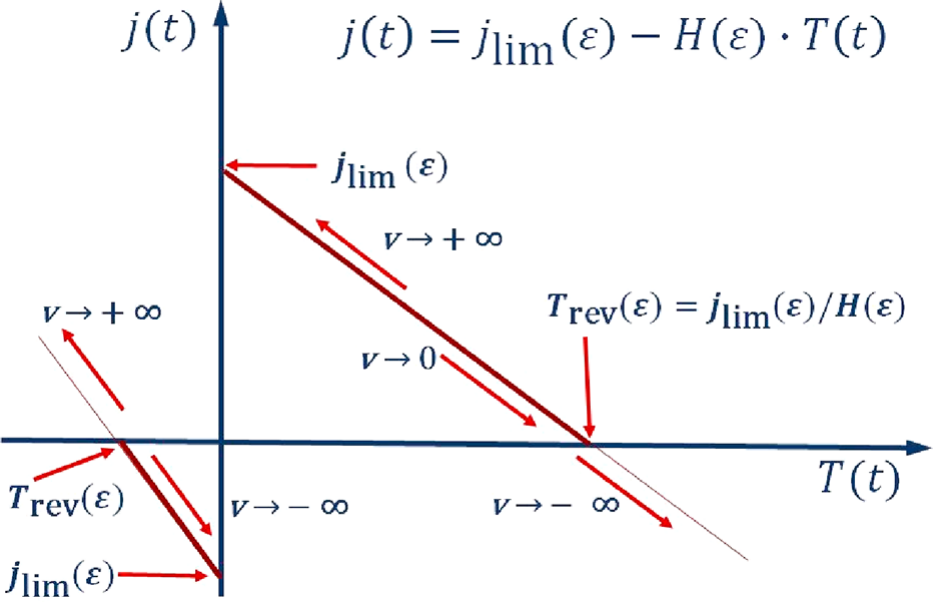
Illustration of eq 12. The two lines represent the two cases when *j*_lim_(ε) < 0 and *j*_lim_(ε) > 0; *v* ≡ d*E*/d*t*.

*Theory—EIS*. Deriving the equations of the impedance functions, based on eq 12, follows the usual way as that of the locally linear electrochemical systems. Assume a small amplitude sinusoidal potential perturbation of the (angular) frequency, ω, on top of a relatively slowly changing potential. Assume that the *Z*(ω) impedance spectrum can be measured in a sufficiently short time, during which the change of the time-averaged potential is negligible, i.e., it may be regarded as a quasi-steady-state potential, *E*_qss_. That is, the potential is *E*(*t*) = *E*_qss_(*t*) + *Ẽe*^iω*t*^, where *Ẽ* is the phasor, i.e., complex amplitude of the sinusoidal signal. In what follows, all tilde-overlined quantities are phasors. This way all the time-dependent quantities denoted by *b* (these are *E*, *j*, *T*) will exhibit a *b*(*t*) = *b*_qss_(*t*) + *b̃e*^iω*t*^ time dependence; the terms *g*(ε), which have time-dependence through that of the potential dependence (these are *j*_lim_ and *H*), will show *g*(*t*) = *g*_qss_(ε) + d*g*/d*E*·*Ẽe*^iω*t*^ time dependence. The subscript qss refers to the slowly changing, quasi-steady-state nature of the *b*_qss_(*t*) and *g* quantities. From now onward, we omit the (*t*) arguments of the *b*_qss_(*t*) quantities and the (ε) arguments of the *g*_qss_(ε) quantities.

Accordingly,
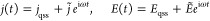
16

As semi-integration and integration of sinusoidal signals are performed by dividing by  and iω, respectively, therefore
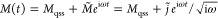
17
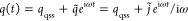
18

Introducing the collective terms *T*(*t*) for *M*(*t*) and *j*(*t*), just as with *T*_qss_ for *M*_qss_ and *q*_qss_, and defining *f*_T_(iω) for the transfer functions  and iω, respectively, we unify eqs 17 and 18 to yield
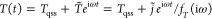
19Substituting eqs 16 and 19 into eq 12 yields the following:
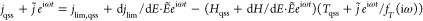
20

This equation holds if both the parts with the qss and those with *e*^iω*t*^ terms simultaneously hold, and the *e*^i2ω*t*^ term can be ignored as it is a product of small amplitude perturbations. For the qss terms, we trivially obtain the same as eqs 12 and 13. From ac terms by rearranging, and expressing the impedance function, *Z*(ω) ≡ *Ẽ*/*j̃*, we get the impedance of a serial two-element circuit:
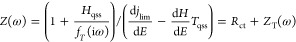
21where *R*_ct_ is the charge transfer resistance:
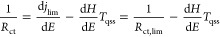
22and *Z*_T_(ω) = *Z*_T_^0^/*f*_*T*_(iω):
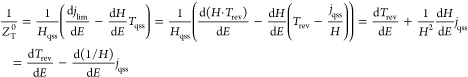
23

The implications of eqs 21–23 are as follows:

(a) According to eq 22, the resistance *R*_ct_ is a PPI function if the second term can be neglected, i.e., *T*_qss_ (*M*_qss_ or *q*_qss_) is small at sufficiently *fast* scans. This statement holds for both (diffredox and surfredox) cases.

(b) According to eq 23, the *Z*_T_^0^ is a PPI function if the second term can be neglected, i.e., *j*_qss_ is small at sufficiently *slow* scans (in principle in equilibrium, when *j*_qss_ = 0). In the diffredox case, the element of *Z*_T_(ω) impedance is a Warburg impedance with *Z*_T_^0^ = σ_W_ = (d*M*_rev_/d*E*)^−1^; in the surfredox case, 1/*Z*_T_^0^ = *C*_redox_ ≡ d*q*_rev_/d*E*.

(c) Equation 21 implies that, whereas both *R*_ct_ and *Z*_T_^0^ do depend on the potential program through *T*_qss_, their ratio
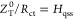
24is a PPI quantity. *H*_qss_ has a straightforward meaning in the related kinetic model; see Table 1. Hence, the main practical conclusion of the present EIS related theory is as follows: for the determination of *k*(*E*) rate coefficients, one has to measure impedance spectra (either sEIS or dEIS) and then to calculate *H*_qss_(*E*) = *Z*_T_^0^/*R*_ct_ (i.e., σ_W_/*R*_ct_ or 1/(*R*_ct_*C*_redox_) for the diffredox or surfredox cases, respectively). *H*_qss_(*E*) has the physicochemical meaning as formulated in Table 1.

*Novelties of the Theory and Its General Nature*. As mentioned earlier, AWV and EIS differ in their potential range and main variable (*t* or ω) of the *E*(*t*) function. These two methods have been used for many decades by different people of different theoretical backgrounds, employing different instrumentation. Accordingly, the two branches of electrochemists performing electrochemical experiments have rarely been in contact with each other. The use of dEIS as in the present theory is an attempt to bridge this gap.

Along with their consequences, eqs 8 to 11 are apparently trivial combinations of three well-known, basic equations of physical chemistry. However, they represent novelties as follows:

(1) We do not attempt to calculate the *j*(*E*) function of a single CV as it was usual in previous theories—since the 1950s—employing exponential potential dependences for the rate coefficients; for numerous examples of these theories, see the textbooks of refs (1 and 2). Instead, we set aside the potential dependence of the rate coefficients and evaluate a set of *j*(*t*) values of different history, e.g., CVs with different scan rates together at the same potential. This is how we can extrapolate to standard surface conditions of kinetics and redox equilibrium at a certain potential. For the extrapolation four equations have been derived, and their main quantities are summarized in Table 2.

**Table 2:** Four Most Important Linear Dependences

Eq. no.	Dependence	Intercept	Slope
12	*j*(*t*) vs *T*(*t*)	*j*_inf_	–*H*
13	*T*(*t*) vs *j*(*t*)	*T*_rev_	–1/*H*
22	1/*R*_ct_(*t*) vs *T*(*t*)	d*j*_inf_/d*E*	–d*H*/d*E*
23	1/*Z*_T_^0^(*t*) vs *j*(*t*)	d*T*_rev_/d*E*	–d(1/*H*)/d*E*

(2) Another novelty is the calculation of the PPI forms of both the large and small signal response functions (AWV and dEIS, respectively) and demonstration of their functional connections, as summarized in Table 3.

**Table 3:** Relation of the Four Important Equations Connecting the Four Measured Quantities [*j*, *T* (*M* or *q*), *R*_ct_, *Z*_T_^0^ (σ_W_ or 1/*C*_redox_)] with the Four PPI Quantities [*j*_inf_, *T*_rev_ (*M*_rev_ or *q*_rev_), d*j*_inf_/d*E*, d*T*_rev_/d*E* (d*M*_rev_/d*E* or d*q*_rev_/d*E*)]

	Charge transfer	Coupling	Diffusion or surface charging
AWV	*j* = *j*_inf_ – *H*·*T*	*H* = *j*_inf_/*T*_rev_	*T* = *T*_rev_ – (1/*H*)·*j*
dEIS		*H* = *Z*_T_^0^/*R*_ct_	

(3) A third novelty of the present Perspective is that it demonstrates the identical mathematical structures of the kinetic equations for two different archetypes (diffredox and surfredox) of electrochemical situations. In addition, as discussed later, there exist additional situations for which analogous models can be set up.

Using the PPI forms is important. Generally speaking, the raw measurement data reflect the properties both of the studied system and of the parameters of the measurement method. Often the first step of data evaluation is when we get rid of the measurement parameters; afterward, the data contain information on the system studied and the physicochemical properties. In other words, data evaluation separates relevant (inherent) and irrelevant (incidental) features.

A PPI function is a state function of *E*. It is not a novelty in electrochemical research. For example, the impedance function is a PPI representation: this function does not “remember” the shape of the potential (or current) function (single sine wave, multisine signal, voltage step, voltage spike, etc.) by which the impedance has been measured.

There are also voltammetry experiments that are often presented in PPI forms. The time-independent polarization curves are obviously PPI ones. CVs of capacitive interfaces are often plotted as so-called dc capacitances, i.e., in a scan-rate-normalized fashion. The “semi-integral electroanalysis” started with the semi-integration of the CVs of reversible redox couples^22^—this procedure yielded hysteresis-free, scan-rate-independent *j*(*E*) plots. These simple situation sets, in the context of the present Perspective, are plotted in Figure 4 as circles; their overlap represents the cases when the current is hindered (but not fully controlled) by diffusion or by the effect of the partial coverage.

**Figure 4:**
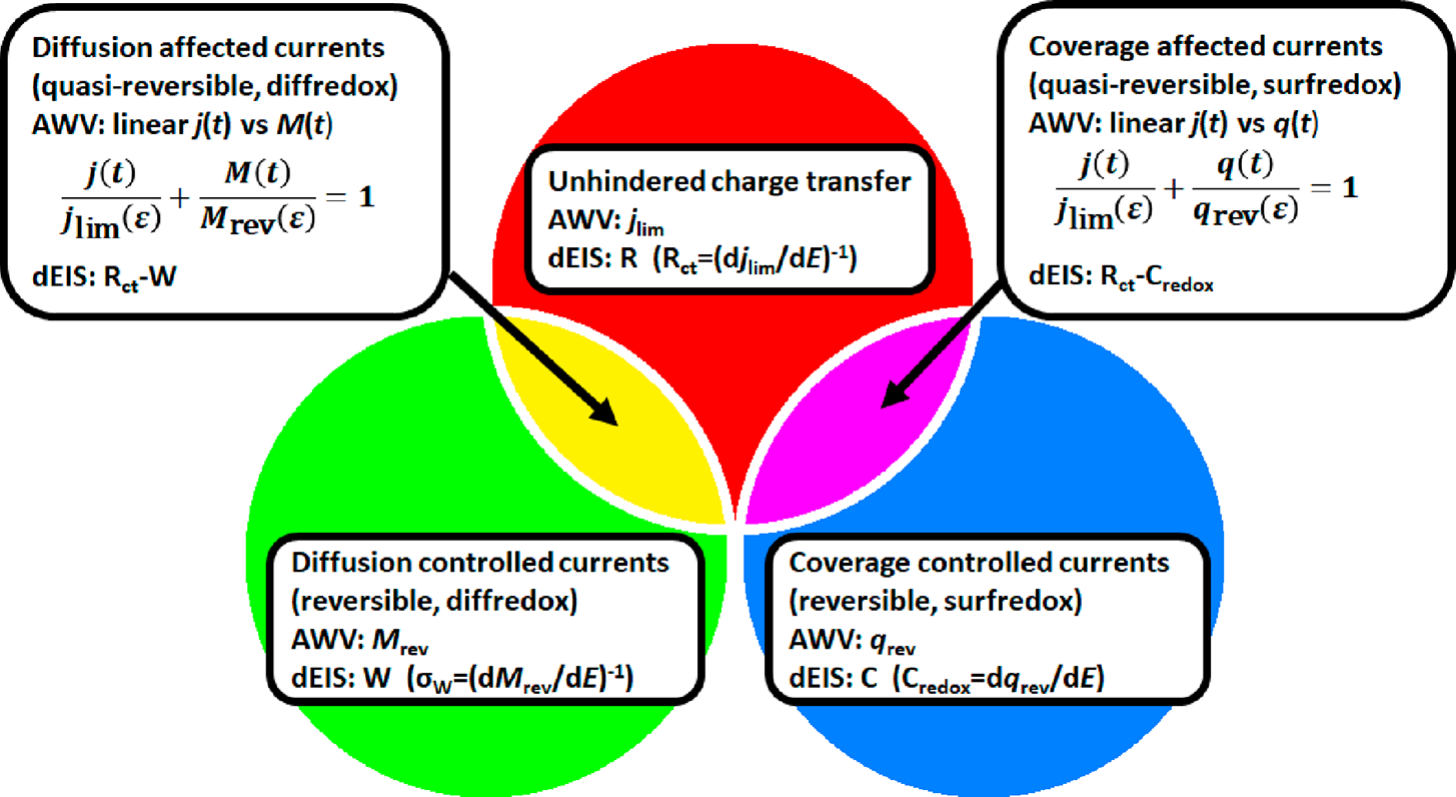
PPI functions and equivalent circuit elements of the simple and coupled situations. The three circles represent the cases where the PPI function is simple (*j*_lim_, *M*_rev_, or *q*_rev_) and the equivalent circuit comprises a single term (R, W, or C). The two overlapping regions correspond to the cases when the current is affected by one type of hindrance. Here the PPI functions can be calculated using eq 15; the equivalent circuit comprises two terms.

The relations of the PPI functions of the present subject, *j*_inf_, *T*_rev_, and their d/d*E* derivatives, are summarized in Table 3. Four points are worth being noted:

(1) Information on charge transfer kinetics can be obtained from data points measured at very short times when we extrapolate to *T* = 0 (cf. the second column of Table 3). In the same vein, information on transport or thermodynamics can be obtained from data points measured at a very long time when equilibrium is already attained and *j* = 0 (cf. the fourth column of Table 3).

(2) Both the intercepts and the slopes of the linear equations of the dEIS are the potential derivatives of those of the AWV. This is how the large-signal and small-signal response functions (AWV and dEIS, respectively, of the given systems) are related to each other through their PPI forms.

(3) The coupling constant *H* can be obtained from PPI functions calculated from voltammetry data, as *H* = *j*_inf_/*T*_rev_. From dEIS data, *H* = *Z*_T_^0^/*R*_ct_. Technically it is much easier and more accurate to use the latter method for the determination of *H*. This is why dEIS measurement is preferred to voltammetry when the determination of rate coefficients is the goal.

(4) One cannot determine individual rate coefficients directly from AWV or dEIS measurements. All that we can obtain is the *H*(*E*) function, which is a combination of rate coefficients (or also diffusion coefficients, cf. Table 1). The decomposition of the *H*(*E*) to individual *k* values is left to a next step.

*dEIS of Adsorption–Desorption Processes*. Besides the diffredox and surfredox cases, there exists the third, intermediate situation, when one species is in the electrolyte bulk, and the other is surface-bound, with the A_red_ ⇄ A_ox_ + *n*e^–^ reaction. This reaction appears as a “real” chemical reaction with a definite transfer of *n* electrons but can also be regarded as an adsorption–desorption process, A ⇄ A_ad_ + *n*e^–^. This is why we call this situation an “adsorption” case. Note that here the meaning of *n* is the (negative of) formal partial charge number,^23^ which might also be a noninteger.

If we apply the simple Langmuirian adsorption–desorption kinetics together with the assumption that no diffusional hindrance prevails, then the rate equation modifies to
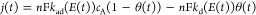
25where *c*_A_ is the bulk concentration of the adsorbate and *k*_ad_ and *k*_d_ are rate coefficients of adsorption and desorption, respectively. Apart from the physical meaning, the complete theory is just the same as that of the surfredox case, with the constants of Table 1 being const_1_(ε) = *j*_lim_(ε) = *n*F*k*_ad_(ε)*c*_A_ and const_2_(ε) = *H*(ε) = *k*_ad_(ε)*c*_A_ + *k*_d_(ε). In this way adsorption–desorption rates can be determined.^18^ As the physical meanings of the equivalent circuit elements given in eqs 22 and 23 are somewhat changed, *R*_ct_ and *C*_redox_ are to be renamed to *R*_ad_ and *C*_ad_, respectively.

To our present understanding, the diffredox, surfredox, and (diffusion-hindrance-free) adsorption situations are the only cases when linear *j*(*t*) – *T*(*t*) relations (and their consequences, like a two-element equivalent circuit for the faradaic impedance) prevail. Nevertheless, provided the potential and time-dependences appear in separate factors of the rate equation, nonlinear *j*(*t*) – *T*_1_(*t*), *T*_2_(*t*), etc. relations can be derived. For example, for diffusion-affected adsorption–desorption, in eq 25 both the surface concentration and the coverage of A (*c*_A_^s^ and θ) are time-dependent; accordingly, also a diffusional impedance appears in the equivalent circuit. Because of the appearance of the *c*_A_^s^(*t*)θ(*t*) product term, any linear equation similar to eq 12 cannot be derived. For this case, a different, more complicated way of analysis, based on three-parameter, nonlinear least-squares fitting, is possible only as shown in ref (21). Further complications, like adsorption–desorption with coverage-dependent rate coefficients, might be handled by multiparameter fitting, as outlined in section 3.5 of ref (21). Most probably, this will be the case when coupling of the adsorption to double layer charging, mixed ion migration, and diffusion are taken into account.^24^ Anyhow, these and further extensions like inclusion of preceding or following chemical reactions would be overcomplicated and are much beyond our present scope.

*Measurement of Rate Coefficients—General Considerations*. To appreciate the above theories, it is instructive to compare them to the other theories for extracting kinetic data from voltammograms and impedance (or related) spectra. Before the advent of computers and data acquisition systems in the laboratories, it was a general practice to do the calculations using the coordinates of characteristic points, e.g., peak currents/potentials. For example, the method of Nicholson of 1965^25^ was widely employed for the determination of charge transfer rate coefficients from the CV peak separation (that is, from distance of the anodic and cathodic peak potentials) using a lookup table. Obviously, the procedure is somewhat inaccurate solely due to the consequence that it is based on two specific points of the whole curve, while all other points of the curves were disregarded. Nowadays CVs are thousands of data points in files within the memory of computers; thus, analysis of the complete curve is feasible. For this reason alone, the modern ways of analysis (including the present one) are superior to those with a few points only: accordingly, evaluation methods by fitting of simulated voltammetry functions to experimental data^26^ are quite common for parameter extraction.

In principle, the AWV measurement and its evaluation are straightforward. One has to measure one or more *j*(*t*) voltammograms with varied scan rates. First the measured *j*(*t*) functions are to be transformed (semi-integrated or integrated) according to the (known or hypothesized) reaction mechanism. Afterward, plotting the *j*(*t*) vs *T*(*t*) points of the same ε potential yields the *j*_lim_(ε) and *T*_rev_(ε). Unless we wish to use it for some electroanalytical purposes, the latter function can be disregarded; *j*_inf_(*E*) as a Tafel plot is to be analyzed further. There is another way to obtain data on kinetics: As it follows from eq 14, the  (for the diffredox case) or the *H* = *k*_c_ + *k*_c_ (for the surfredox case) parameter combination can be directly obtained by calculating *j*_inf_/*T*_rev_ as a function of potential. Alternatively, from dEIS measurement, *H* can be directly obtained by the calculation of *H*_qss_(*E*) = *Z*_T_^0^/*R*, cf. eq 24.

The next step in the diffredox case is the determination of the diffusion coefficients. They are independent of potential and can be obtained through special *M*_rev_(| *E* ≫ *E*_1/2_ |) measurements (like measuring Cottrell transients). Finally, to separate the anodic and cathodic rate coefficients, we have to assume a certain (typically exponential) potential dependence of them.

*Measurement of Rate Coefficients—Technical Issues of the dEIS Measurement*. There is a subtle benefit to the PPI transformations: The *j*_inf_(*E*) and *T*_rev_(*E*) plots provide a self-consistency check of the analysis. These plots should be, in principle, hysteresis-free if its points are calculated separately from the forward and backward scans; the *M*_rev_(*E*) or *q*_rev_(*E*) functions should exhibit the sigmoid shape, and the potential dependence of *j*_inf_(*E*) should be more-or-less compatible with the Butler–Volmer equation. This feature, the demonstration of self-consistency, is a big boon that we get when we apply the present theory for data analysis.

There exist two usual complicating effects when we analyze voltammetric curves: the IR drop, due to the nonzero solution resistance, and the double layer charging. EIS measurements, simultaneously done with CV (i.e., dEIS), contribute to higher accuracy; namely, if we determine *R*_s_ and *C*_dl_(*E*) from the spectra, then the scales of the potential and current density of the voltammograms can be easily corrected by subtracting the IR drop (=*j*·*R*_s_) and the charging current (=*C*_dl_(*E*)·d*E*/d*t*), respectively. Actually, this is the point where the big advantage of dEIS is apparent over the traditional, simple voltammetry and EIS measurements: dEIS provides not only the information on kinetics (cf. eq 12) but simultaneously the correction factors *R*_s_ and *C*_dl_(*E*).

For the classical impedance measurements, we always assume the steady state of the system; for dEIS, we assume the quasi-steady state of quantities (with subscript qss). Evidently, this condition holds for slow scans with high-frequency impedance measurements and does not hold for fast scans with low frequencies. In general, the quasi-steady-state condition applies for all quantities appearing in the kinetic equations for concentrations, potentials, and currents. For example, surface concentration change due to the quasi-steady-state current during one impedance measurement should be much smaller than the one corresponding to the perturbation amplitude, as was emphasized in ref (27). An important parameter, the maximum scan rate, *v* = d*E*/d*t*, depends on how fast we can measure the impedance spectra, which in turn depends on many technical details of the impedance spectrum measurement. As a rule of thumb, we can say that the spectrum measurement is correct if the Δ*E* potential change during two subsequent spectrum measurements is smaller than *RT*/*F* (≈27 mV). For the published dEIS setups of refs (10−14), the Δ*E* = *v*Δ*t* potential spacing is typically on the order of magnitude of 1–10 mV.

*Measurement of Rate Coefficients—An Example for the dEIS Measurement*. We have constructed a dEIS measurement setup comprising a potentiostat and a home-built data acquisition system, DAQ, and have written the appropriate software. The DAQ provided the multisine voltage and measured four voltages with 50 kHz frequency simultaneous sampling. Two input channels served for sampling the appropriately low-pass-filtered signals of potential and current, and with the two other channels, the (high-pass-filtered and amplified) high-frequency signals of potential and current were measured. The minimum frequency of the spectra depended on the scan rate *v*, as *f*_min_ = 3*v*/Δ*E* with constant Δ*E* = 16 mV (typical dEIS parameters were *v* = 100 mV/s, *f*_min_ ≈ 20 Hz). Accordingly, altering *v* was always accompanied by changing the *f*_min_. By checking the spectra with a Kramers–Kronig test program, we found no sign of any low-frequency distortion. The measurement series below required (with the necessary breaks) altogether less than two minutes. For other technical details, see ref (15).

To demonstrate the quality of that setup, testing was done with a well-known electrochemical system of a ferro/ferricyanide couple on a noble metal electrode, gold. The interfacial reaction on this system is considered to be a simple, fast, one-step, one-electron “outer sphere” electron transfer with little if any complications, apparently ideal for demonstrating diffusion-affected (quasi-reversible) voltammetry and impedance properties. For the history of experiments on this or similar systems, see section 2.1 of ref (28); one should highlight the precise EIS measurements of Randles 70 years ago^29^ yielding standard rate coefficients around 0.1 cm/s and those of Peter,^30^ which demonstrated the proportionality of the rate coefficient on the K^+^ concentration of the electrolyte. There appears to be some complication due to the formation of a surface layer^31^ which can be traced back to the strong adsorption of ferricyanide.^32^ The surface layer slows electron transfer; this is why the dEIS measurements should be carried out as fast as possible.

Our measurements and results are described in ref (28). We repeat here only the most important features.

The CV parts of dEIS measurements of 10 mM ferrocyanide in 0.5 M KF base electrolyte on polycrystalline gold electrode for three different scan rates are shown in Figure 5a as continuous lines. The circles are at the potential locations of the measured impedance spectra.

**Figure 5:**
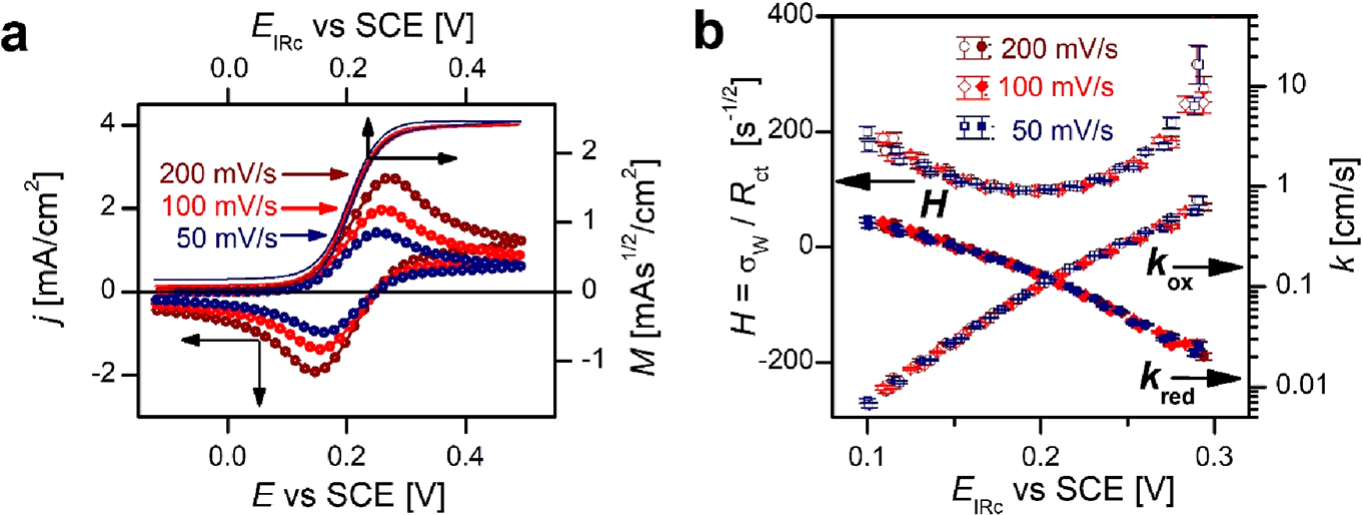
Evaluation of dEIS measurements on planar Au electrode in 0.5 M KF solution containing 10 mM K_4_⌈Fe(CN)_6_⌉ to yield charge transfer rate coefficients. (a) CVs of scan rates are indicated; points mark the potentials around which impedance spectra have been measured. The sigmoid curves are the semi-integrated CVs (their scale is on the right ordinate) vs the IR-corrected potential, *E*_IRc_ (abscissa on the top). (b) The *H* = σ_W_/*R*_ct_ ratio, as calculated from the dEIS spectra and the rate coefficients as a function of the IR-corrected potential, *E*_IRc_.

The semi-integrated CV curves, after performing the IR correction of the potential scale (using the *R*_s_ determined from the impedance spectra, see later), also shown in Figure 5a, are practically hysteresis-free and coincide—indicating that the charge transfer is very fast, and rate coefficients cannot be determined using eq 12. However, from the plateau height, the diffusion coefficient of the ferrocyanide ions can be calculated.

The impedance spectra could be fitted well with the impedance function of the Randles circuit. From the point of view of charge transfer kinetics, the σ_W_/*R*_ct_ ratio is important—σ_W_ and *R*_ct_ could be determined precisely at potentials close to the CV peaks. The values *C*_dl_ were found to be approximately the same as that of gold in the base electrolyte; and finally, *R*_s_ was used for the correction of the potential scale.

The  quantity as a function of the IR-corrected potential is plotted in Figure 5b. Assuming that *D*_ox_ = *D*_red_ and taking into account that for a single-step, one-electron charge transfer *k*_ox_(*E*)/*k*_red_(*E*) = exp(F(*E*^0^ – *E*)/*RT*),^33^*k*_a_(*E*) and *k*_c_(*E*) can be separately plotted, as also shown in Figure 5b. The cross point of these curves yields the equilibrium rate coefficient 0.11 cm/s.

The σ_W_/*R*_ct_, and hence also *k*_ox_ and *k*_red_, points of the forward and backward scans are along the same single curve, for all scan rates. This is a self-consistency feature. If the σ_W_/*R*_ct_ values, and hence also the rate coefficients, decrease in subsequent scans, then it indicates the spoiling of the electrode surface (probably through chemical reactions with the electrode surface). As the *k*_a_(*E*) and *k*_c_(*E*) rate coefficients are outputs of the measurements, we stress the importance of the bending of the log(*k*_a_(*E*)) and log(*k*_c_(*E*)) curves: it clearly indicates that the charge transfer coefficients do depend on the potential.

*Summary, Conclusions and Future Perspectives*. We recently elaborated a couple of theories with the aim of determining the rate coefficients of simple electrochemical reactions from voltammetry curves and impedance spectra. Three reaction schemes were considered: diffusion-affected charge transfer, charge transfer of surface-bound species, and adsorption. The theories yielded equations by which the voltammograms can be transformed to potential-program invariant forms, allowing an easy calculation of the rate coefficients; similar equations have been derived for the potential dependence of equivalent circuit parameters obtained from dEIS measurements.

In this Perspective, the above theories are condensed into a unified, two-part mathematical model. In the first part, we obtain linear equations from which we can extrapolate voltammetric currents measured at the same potential to infinite transport rate or zero coverage, cf. eq 12. The extrapolated currents are independent of the potential program by which they have been measured.

In the second part of the theory with a similar line of thought, we analyzed the properties of the high-frequency impedance spectra while a slow scan voltammetry measurement is running. The fast high-frequency spectrum measurement acts as if a snapshot were taken on the electrode, which is in a quasi-steady state. Two features are noteworthy: First, although the equivalent circuit parameters differ from those at a constant potential, extrapolation to zero scan rate is possible, cf. eqs 22 and 23. Second, certain combinations of equivalent circuit parameters are scan-rate independent, cf. eq 24. Consequently, for example, rate coefficients of diffusion-affected charge transfer reactions can be measured by dEIS with relatively fast scan rates, that is, in a short time.

The above-mentioned theories, just as their present unified version, cover an important part of the set of simple reaction mechanisms. However, their application is suggested only if the linear relationship between the current and its transform can be justified. These are typically the cases when *R*_s_ and *C*_dl_ are not counted and the equivalent circuit consists of two elements only. More complicated reaction schemes would lead to problematic transformations of voltammograms and with more than two impedance elements in the equivalent circuit.

We recommend the use of the present theories for teaching electrode kinetics (in the classroom) and for the evaluation of measurement data, in particular, of dEIS results (in the laboratory). We propose the theory for teaching because of the following three reasons:

(1) It is easy to decouple diffusion and charge transfer in the case of diffusion-affected charge transfer reactions just as adsorption isotherm and adsorption kinetics; the separation leads to simple linear equations like eq 12 or 15.

(2) Using the linear equations one can obtain potential-program invariant forms of the measured data. That is, the essential physicochemical quantities are separated and the eventual technical parameters are eliminated. In this way, we carry out standardization of the measurement results.

(3) The theory connects CV (or AWV) and EIS, through a similar formalism. In this way, a bridge between large and small signal responses is created.

We recommend the theory also for those doing research in the laboratory for the following three reasons:

(1) The high speed of dEIS makes it possible to perform fast measurements of kinetics or the double layer on freshly prepared (e.g., annealed) electrodes with reduced danger of the contamination or transformation of the electrode surface.

(2) Since there has been no *a priori* assumption involved on the potential dependence of the rate coefficients, this dependence appears as an output quantity when measurement data are analyzed. This feature is important when the potential dependence of rate coefficients is studied.

(3) Transparency of the data evaluation is improved. dEIS is better than plain voltammetry because step-by-step evaluation and plotting reveal the not self-consistent features. The impedance measurements also provide the correction factors.

The theories summarized in this Perspective open new routes for the determination of rate coefficients of various hindered charge transfer electrode reactions.

## List of Symbols and Acronyms

PPI: potential-program invariant (representation, function)
CV, AWV: cyclic and arbitrary waveform voltammetry/voltammogram
EIS: electrochemical impedance spectroscopy/spectrum
sEIS: static EIS
dEIS: dynamic EIS
diffredox: diffusion affected/controlled charge transfer
surfredox: charge transfer reaction when both species are surface-bound
*n*: number of electrons taking part in the redox reaction
*F*: Faraday’s number
*R*: universal gas constant
*T*: temperature
*t* [s]: time
*E* [V]: electrode potential (in general)
*v* [V/s]: scan rate
*j* [A/cm^2^]: current density
ε[V]: electrode potential, in the context of eqs 4–15
*k*_a_ [cm/s]: anodic rate coefficient
*k*_c_ [cm/s]: cathodic rate coefficient
*k*_ad_ [cm/s]: adsorption rate coefficient (adsorption case)
*k*_d_ [cm^3^/mol/s]: desorption rate coefficient (adsorption case)
*c*_red_ [mol/cm^3^]: concentration of the red species in the solution bulk (diffredox case)
*c*_ox_ [mol/cm^3^]: concentration of the rox species in the solution bulk (diffredox case)
*c*_red_^s^ [mol/cm^3^]: concentration of the red species in the vicinity of the surface (diffredox case)
*c*_ox_^s^ [mol/cm^3^]: concentration of the ox species in the vicinity of the surface (diffredox case)
*c*_A_ [mol/cm^3^]: concentration of the adsorbate in the electrolyte bulk (adsorption case)
*c*_A_^s^ [mol/cm^3^]: concentration of the adsorbate in the vicinity of the surface (adsorption case)
*D*_red_ [cm^2^/s]: diffusion coefficient of the red species (diffredox case)
*D*_ox_ [cm^2^/s]: diffusion coefficient of the ox species (diffredox case)
θ(*t*): coverage of the reduced species (surfredox case) or of the adsorbed species (adsorption case)
Γ_0_ [mol/cm^2^]: surface concentration of the redox system (surfredox case)
*H*(ε) [1/s]: parameter combination, see Table 1
*j*_lim_(ε) [A/cm^2^]: current density at potential ε when no hindrance prevails
*M*(*t*) [A√s/cm^2^]: semi-integral of the current density, cf. eq 5
*q*(*t*) [C/cm^2^]: surface charge, cf. eq 9
*T*(*t*): collective term for *M*(*t*) and *q*(*t*)
*M*_rev_(ε) [A√s/cm^2^]: semi-integral of current density, in reversible diffredox case
*q*_rev_(ε) [A·s/cm^2^]: surface charge, in reversible surfredox case
*T*_rev_(ε): collective term for *M*_rev_(ε) and *q*_rev_(ε)
ω [1/s]: (angular) frequency of the sinusoidal perturbation
*Z*(ω) [ohm × cm^2^]: impedance at frequency ω
*T̃*, *Ẽ*, *j̃*, *b̃*, *g̃*: tilde-overlined quantities: phasors at frequency ω
*E*_qss_, *j*_qss_, *b*_qss_, *g*_qss_: subscript qss indicates quasi-steady-state component of the quantities
*f*_T_(iω): collective term for transfer functions and iω
*R*_ct_ [ohm × cm^2^]: charge transfer resistance
σ_W_ [ohm × cm^2^/√s]: coefficient of the Warburg impedance (diffredox case)
*C*_redox_ [F/cm^2^]: redox capacitance (surfredox case)
*R*_ad_ [ohm × cm^2^]: adsorption resistance (adsorption case)
*C*_ad_ [F/cm^2^]: capacitance (adsorption case)
*Z*_T_^0^: collective term for σ_W_ and 1/*C*_redox_
*C*_dl_ [F/cm^2^]: double layer capacitance
*R*_s_ [ohm × cm^2^]: series/solution resistance
